# The International Medical Congress

**Published:** 1876-09

**Authors:** 


### Proceedings of Medical Societies.

### THE INTERNATIONAL MEDICAL CONGRESS.

The Congress was opened on Monday, Sept. 4th, at twelve o'clock, by Prof. Samuel D. Gross, M. D., LL. D., D. C. L., Oxon. After prayer by the Right Rev. Wm. Bacon Stevens, Bishop of Pennsylvania, Prof. Gross delivered an address of welcome, which we are happy to be able to give in full, as follows:

"My colleagues have confided to me, as the President of the Centennial Medical Commission, the agreeable and honorable duty of opening this International Medical Congress, so long the object of their solicitude and earnest labor. In their name, then, as well as my own and that of the entire medical profession, whose great heart this day throbs in unison with ours, I extend to you our right hand, and bid you a thrice cordial welcome to the City of Brotherly Love. The occasion which has brought us together this morning is one of no ordinary kind; it is one also which has been long and, I may say anxiously anticipated. I might, perhaps, seem ungracious if I were to tell you how much time and labor have been bestowed by the commission through its Committee of Arrangements upon the organization of the Congress; how often they met to devise plans and to interchange views; how earnestly and thoughtfully they performed their work; in a word, how faithful and conscientiously they discharged the great trust confided to them by the different medical bodies of the city and county of Philadelphia, in which the Congress originated nearly two years ago. Not a little embarrassment often attended their progress, and it was, therefore, not without a profound sense of relief, such as a weary traveller may be supposed to experience at the end of a long and tedious journey, when we found that our task was finally brought to a successful close. If the organization is less complete than to some of you it may seem to be, no blame will, I am sure, be ascribed to the commission on account of any shortcomings. There might, possibly, have been wiser and more experianced heads at work; but warmer hearts, or more conscientious men never were, I venture to affirm, engaged in a noble enterprise. Such, then, as the work is, we cordially submit it to your consideration, satisfied that it will be accepted by you in the same kindly spirit in which it is tendered, and that any deficiencies that may mar its character may be duly rectified by your superior wisdom.

It is at all times a source of gratification to welcome friends, especially when they are united by the bond of a common brotherhood, or an identity of interests; but on this occasion, so pregnant with important events, the feeling is vastly heightened by the fact that we have assembled around us brethren not only from every section of this great continent, but from various foreign climes—from Europe, the far East, from Japan and China, the islands of the Pacific, South America, Mexico, and the West Indies, and, I had almost said, from every country in the world. The invitations sent out by the commission cover every prominent medical society and every distinguished medical man in the four quarters of the globe. The object was to bring together representative men from all nationalities to participate in our proceedings, and to afford us the benefit of their wisdom, and the results of their experience and scientific investigations. If all these, or even a respectable minority of these representative men could have been here, what a glorious spectacle would be presented in this hall this morning! Men laying aside for a while their ordinary pursuits, crossing vast continents and perilous seas, congregating to unite with us in celebrating our first Medical Centennial, in interchanging cordial salutations, in deliberating upon the best means of promoting the holiest and dearest interests of our profession, and in laying their contributions—the accumulations of years of study and observation—upon a common altar for the common good! In its wide range, the present Congress is without a parallel. Similar bodies have repeatedly met, but none on so grand a scale or with such a cosmopolitan outlook.

In organizing the Congress the commission may have been guilty of undue partiality towards their own country. Perhaps such a tendency was, after all, natural. However this may be, certain members had an irresistible desire to show the world what the century, since the establishment of our independence as a free and sovereign people, has accomplished for scientific medicine. For this purpose topics illustrative of the progress and present condition of the different branches of medicine in the United States have been assigned to gentlemen of acknowledged rank in the profession in different sections of the Union. These exercises will, it is believed, add greatly to the interest of the occasion. Time was when we had no medical literature—no medical science—when we were utterly helpless, and wholly dependent upon the aid derived from our European brethren, especially the English, whose language, practice, and habits we made our own. The poverty of the country in these respects cannot be better illustrated than by the fact that we had no native works on medicine and the collateral sciences until after the commencement of the present century. Many of you will recall the words of the great English lexicographer who, in 1769, in speaking of the American colonies, exclaimed, 'Sir, they are a race of convicts, and ought to be thankful for anything we allow them short of hanging.' The Abbe Raynal, writing in the latter part of the last century, declared that America had not produced a single man of genius; and the exclamation of a celebrated Scotch reviewer, uttered at a more recent period, 'Who reads an American book, who goes to an American play, or who looks at an American picture?' is still fresh in the memory of many of the present race of men. The discourses which will be delivered before you on the progress of American medicine will serve to show that the profession of the United States has earned for itself an enviable reputation, and that it is fully abreast with all the other pursuits that adorn the human mind and shed luster upon the scientific character of the nation. They will serve to show that we have passed the period of medical provincialism, and that we stand upon a loftly platform, to which we need not be ashamed to invite the representative men of the profession of foreign countries, however illustrious, or however far advanced in the arts of civilization.

The different sections, organized by the commission, must speak for themselves. It is in them that the work of the Congress is mainly to be done, where the interchange of scientific ideas is to be effected, and from which the meeting is to derive its chief glory as an international body of scientific and enlightened men.

It will be recollected that attempts have been made at different times to establish uniformity of scientific nomenclature, weights, measures, and records of disease, for the medical profession in all parts of the civilized world. The plan, if carried out, cannot fail to advance in an eminent degree, the interests of medical science; and I am happy to state that it is proposed to discuss the subject fully in one of the sections.

We are upon the threshold of a new century. One hundred years have passed away since the grand old bell upon Independence Hall announced to the world the birth of a new nation, and liberty not only to our own citizens but to all the nations of the earth. The new century that has just elapsed was the most wonderful in all that pertains to human progress, to discovery, to invention, to improvement, to refinement and intellectual culture; in a word, to all that ennobles and exalts human nature in its various aspects and phases, that has been vouchsafed to man since God said, 'Let there be light.' The science of medicine has been completely revolutionized within our own day. The saying, 'Old things have passed away, behold all things are new,' has literally been fulfilled. The microscope, chemical analysis, clinical observation, and experiments upon the inferior animals, are leading on the medical mind with wondrous velocity in the pursuit of knowledge, and adding daily new facts to our stock of information far beyond what the wildest fancy could have conceived of even a third of a century ago. Dogmatism, once so dominant in the schools, has ceased to exist, and no unacknowledged theories are any longer received by the scientist. Facts, resting upon the broad basis of observation and experiment, repeated and varied in a thousand ways, alone, are relied upon as worthy of acceptance and as safe guides in practice. Hippocratic medicine is the order of the day. Everything bows before its divine behests.

In every corner of the habitable globe, penetrated by the light of civilization, busy, active minds, endowed with high culture, and actuated by the noblest resolves, are at work, exploring the mysteries of disease, and devising means or methods of treatment, for the relief of suffering, and the prolongation of life. The busy bee was never more industriously engaged in gathering honey from the flower of the field than the modern physician is in gathering knowledge at the bedside of the sick, and garnering it for future use. Much of what is considered by many as established must be reviewed in the light of modern science; new avenues must be opened, and the ball, composed of myriads of threads more delicately formed than any ever spun by Penelope, must be pushed onward and upward by the united efforts of the medical profession in all parts of the world. How far the Centennial International Congress shall promote these desirable objects time alone can determine. It may safely be predicted that, if it do not fulfil all the promises of hope that have been formed of it, it will accomplish a vast deal of useful work, and thus afford the world an earnest of its interests in the advancement of scientific medicine and in international unity. Science can have no higher mission than that of strengthing the bonds and securing the co-operation of its votaries in various parts of the globe, assembled to deliberate upon everything calculated to promote its holiests interests.

Among the many objects of an International Congress, not the least is the interchange of kindly feelings on the part of its members, the formation of new friendships and the cementing of old ties. It is well that men of different nationalities should occasionally come together, to look at one another, and to see how they stand in public estimation, as well as their own; what the world thinks of them, and what they think of the world; what they have done to further the interests of scientific progress, to lighten the burdens of human suffering, and to extend the boundaries of human happiness. All these and many other things which need not to be here specified, are objects well calculated to engage attention on such occasion.

It need hardly be added that the medical profession and the citizens of Philadelphia will do all they can to make your time pass pleasantly, as well as profitable, during your sojourn among us. Cards of invitation will be issued to you to inspect the various institutions of interest in and around the city; and, after the work of the Congress is over, the International Exposition will no doubt claim, as it assuredly deserves, the earnest attention of every member of this body. And now that the labor of the Centennial Medical Commission is complete, it only remains for the Congress, which I now declare open, to perfect its organization by the election of its own officers.

It has often occurred to me that if these international reunions were more frequent and more largely attended, they would be a vast deal more serviceable in preventing wars and international misunderstandings than any arbitrations that could be inaugurated for the settlement of international difficulties. Much of the pleasant feeling at present existing between the United States and Europe is due to the enlarged intercourse which has been going on since the invention of steam navigation between the peoples of those countries, and the consequent interchange of hospitality and courtesy between the two countries. I hope, therefore, that this may be only one of the many such reunions on this side of the Atlantic.

The Chair then announced the following committee on nomination, to select officers for the Congress:

Mr. William Adams, London; Prof. Engelsted, of Copenhagen; Prof. Hüter, of Greifswald; Prof. Rudnew, of St. Petersburg; Dr. J. A. Grant, of Ottawa, Canada; Dr. Henry I. Bowditch, of Boston; Prof. L. A. Dugas, of Augusta, Georgia; Prof. J. T. Hodgen, of St. Louis; Prof. Christopher Johnston, of Baltimore; Prof. Austin Flint, Sr., of New York; Dr. W. S. W. Ruschenberger, U. S. Navy; Dr. Joseph R. Smith, U. S. Army; Dr. Edwin M. Snow, of Providence, R. I.

While the nominating committee were consulting, Prof. Austin Flint, of New York was introduced, and gave the address on medicine, of which the following is an abstract:

He alluded to the influence exercised by the medical men of the Revolutionary period, to the commencement at that early date of a medical literature, and to the formation of medical schools. The war, which called forth the medical energies of the colonies, for a time, arrested the progress of medical schools and medical teaching, but it resulted in the development of a body of medical men who had added to the theoretical a vast amount of that which was practical, obtained in army practice.

The dominant medical minds were in New York, Philadelphia and Boston. In Philadelphia the Philadelphia Medical College was founded, Franklin being one of its most active promoters. In 1791 this school was merged into the Medical Department of the University of Pennsylvania, which was formed after the model of the school of Edinburgh. Philadelphia inaugurated medical teaching in America, she was then and has been since the medical centre. The teachers in the Philadelphia school were all graduates of Edinburgh, and very naturally clung to the views they had received there.

The views of Cullen were generally adopted, and for a time prevailed, especially in Hospital practice. His works had been edited by Rush, at that time the leading medical man in America. Cullen's system was followed by others, each prominent lecturer or writer seeking to evolve some system which should embrace the whole science of medicine, this era, in fact, may be styled that of medical systems.

At the close of the first quarter of the nineteenth century, there were more than twenty medical schools in the United States, six of these were in New York, Philadelphia and Boston, the balance were provincial, these latter supplied the place of the more expensive and distant colleges to students who could not afford the expense of city tuition, they developed study and inquiry among physicians in their vicinity, and trained many of the teachers in the metropolitan schools. There were at this time twenty Medical Journals. The important agencies in the growth of medicine were the views of Cullen, the pathologist of greatest strength at close of the last century, following whom was Rush, Good and Brown, the latter of Edinburgh, the opponent of Cullen. In 1801 Seaman, of New York, began to vaccinate; in 1817 the Pharmacopœia of 1820 was projected; in 1846 anæsthetics were first used in Boston.

The appearance of American translations of foreign works and the publication of Horner's work upon pathological anatomy, of a later of one by Gross upon the same subject, were mentioned as adjuncts to the advance of medicine in America.

The introduction of auscultation and percussion in diagnosis of diseases of the heart and lungs, the publication of the works of André, Hope, Stokes, and the writings of Holmes, James Jackson, Bowditch and Gerhard were alluded to. The Clinical reports of Dr. Bright, and the advance of diagnosis of diseases of the kidneys, to which they gave rise, were alluded to.

In 1828, the *American Journal of the Medical Sciences* was first issued, its work in medical literature was highly spoken of. Brussaisism was the last of the "isims," it was succeeded by the "pathies" of to-day. The works of John Eston Cook, who believed that congestion of the liver was the foundation of all disease, were next mentioned, the heroic doses of calomel administered in his day were illustrated by an amusing story of a farmer who imagined he had discovered a mine of quicksilver, but was disappointed to find that the metalic mercury which he had found was on the burial lot of a number of patients treated by mercury.

The self-limited nature of disease as advocated by Bigelow in his work, produced a change in the treatment of diseases, polypharmacy and heroic doses fell into disuse, physicians began now to doubt their powers of *curing* diseases and began to watch and endeavor to assist nature.

The characteristics of the last quarter of the century were stated to be increased attention to histology and pathology, the influence of German medical literature, and the increasing tendency to go abroad to study medicine. After a brief reference to the advantages to be gained by an international copyright law, to the advanced position of our medical literature, Dr. Flint said the chief characteristic of our medical schools is their practical teaching. He dwelt, in conclusion, upon the Code of Ethics and its value, and to the elevated social and public position of the profession in this country.

Dr. Gross called attention to the fact that Dr. Flint had with great modesty omitted all mention of his own contributions to medical science.

On motion of Dr. James P. White a vote of thanks was tendered to Drs. Gross and Flint, and copies of their addresses asked for publication.

The Committee on Nominations then reported the following names for officers of the Congress:

*President:* Professor Samuel D. Gross.

*Vice-Presidents:* Prof. Paul F. Eve, of Tennessee; Mr. Joliffe Tuffnell, of Ireland; Drs. W. L. Atlee, of Pennsylvania; C. Lange, of Denmark; J. B. Johnson, of Missouri; F. Semeleder, of Austria; Dr. Hunter McGuire, of Virginia; Dr. Johan Hjort, of Norway; Dr. T. G. Richardson, of Louisiana; Dr. Wm. H. Hingston, of Canada; Prof. J. P. White, of New York; Dr. H. Miyake, of Japan; Prof. Nathan R. Smith, of Maryland; Prof. Rudnew, of Russia; Dr. Joseph M. Toner, of Washington; Prof. Hueter, of Pomerania; Dr. G. L. Collins, of Rhode Island; Dr. R. F. Hudson, of Australia; Dr. Henry Gibbons, of California; Dr. P. Debasieux, of Belgium; Dr. N. S. Davis, of Illinois; Mr. William Adams, of England; Dr. L. Dugas, of Georgia; Prof. A. R. Simpson, of Scotland; Dr. J. K. Bartlett, of Wisconsin.

*Honorary Vice-Presidents:* Surgeon-General J. K. Barnes, U. S. Army; Surgeon-General Joseph Beale, U. S. Navy.

*Treasurer:* Dr. Caspar Wistar, of Pennsylvania.

*Secretary General:* Dr. I. Minis Hays, of Pennsylvania.

*Secretaries of Meetings:* Drs. W. B. Atkinson, Richard J. Dunglison, R. A. Cleemann, W. W. Keen, R. M. Bertolet.

Officers of Sections. Medicine. *Chairman.*—Alfred Stillé, M. D., Philadelphia; *Vice-Chairmen.*—R. P. Howard, M. D., Canada; J. J. Woodward, M. D., U. S. A.; *Secretary.*—J. Ewing Mears, M. D., Philadelphia.

Biology. *Chairman.*—John C. Dalton, M. D., New York; *Vice-Chairmen.*—Austin Flint, Jr., M. D., New York; F. W. Campbell, M. D., Canada; *Secretary.*—James Tyson, M. D., Philadelphia.

SURGERY. *Chairman.*—Prof. Joseph Lister, Edinburgh; *Vice-Chairmen.*—J. A. Grant, M. D., Canada; John Ashhurst, Jr., M. D., Philadelphia; *Secretary,*—John H. Packard, M. D., Philadelphia.

DERMATOLOGY AND SYPHILOLOGY. *Chairman.*—James C. White, M. D., Boston; *Vice-Chairmen.*—S. Engelsted, M. D., Copenhagen; Edward Shippen, M. D., U. S. N.; *Secretary.*—A. Van Harlingen, M. D., Philadelphia.

OBSTETRICS. *Chairman.*—Robert Barnes, M. D., London; *Vice-Chairmen.*—Prof. Alex. R. Simpson, Edinburgh; W. H. Byford, M. D., Illinois; *Secretary.*—William Goodell, M. D., Philadelphia.

OPHTHALMOLOGY. *Chairman,*—R. Brudenell Carter, F.R.C.S., London; *Vice-Chairmen.*—Wm. Thompson, M. D., Philadelphia; Henry W. Williams, M. D., Boston; *Secretary.*—John Green, M. D., St. Louis.

OTOLOGY. *Chairman.*—Clarence J. Blake, M. D., Boston; *Vice-Chairman.*—A. H. Buck, M. D., New York; *Secretary.*—H. N. Spencer, M. D., St. Louis.

SANITARY SCIENCE. *Chairman.*—Stephen Smith, M. D., New York; *Vice-Chairman.*—J. S. Billings, M. D., U. S. A.; *Secretary.*—E. M. Hunt, M. D., New Jersey.

MENTAL DISEASES. *Chairman.*—John P. Gray, M. D., New York; *Vice-Chairmen.*—E. Grissom, M. D., North Carolina; I. Ray, M. D., Philadelphia; *Secretary.*—Walter Kempster, M. D., Wisconsin.

COMMITTEE ON PUBLICATION, WITH POWER TO SELECT CHAIRMAN AND EDITOR. John Ashhurst, Jr., M. D., Philadelphia; R. J. Dunglison, M. D., Philadelphia; Wm. Goodell, M. D., Philadelphia; James H. Hutchinson, M. D., Philadelphia; Caspar Wister, M. D., Philadelphia.

The Congress then adjourned.

At two, P. M., a public luncheon was served to the members of the Congress, in the basement of the University, and at three, P. M., the sections assembled for work. In most of the sections we can only give the title of the papers read. They were as follows:

In Section I. MEDICINE.—Typho-malarial Fever; is it a Special Type of Fever? Reporter, J. J. Woodward, M. D., Surgeon U. S. Army; in Section II. BIOLOGY.—Microscopy of the Blood. Reporter, Christopher Johnston, M. D., Professor of Surgery in the University of Maryland; in Section III. SURGERY.—Autiseptic Surgery. Reporter, John T. Hodgen, M. D., Professor of Surgical Anatomy and Clinical Surgery in the St. Louis Medical College.

His conclusions may be classed under the following four heads:

I. Putrefaction may and does occur in the solids and liquids of the body both with and without the direct contact of germs borne in the air or water.

II. Putrefaction of the solids and liquids of an open wound may in many cases be prevented if the contact of living germs with the surface is not permitted, or by destroying their vitality after contact with it.

III. It is possible that the living solids and liquids of the body may be so altered that they shall not furnish the conditions necessary to putrefaction.

IV. Practically the conditions to be met in preventing putrefaction are so difficult that in many cases it is impossible to comply with them. Yet, even partial success is eminently worthy of our best efforts.

This paper, into the details of which we cannot go at the present, was discussed by Dr. Hewson of Philadelphia, who some time since brought foward the subject of earth dressing, Drs. Hamilton and Weir of New York, Dr. Caniff of Toronto and Prof. Lister of Edinburgh.

In Section IV, Dermatology and Syphilology, the paper was upon Variations in Type and in Prevalence of Diseases of the Skin in Different Countries of Equal Civilization. Reporter, James C. White, M. D., Professor of Dermatology in Harvard University.

The programme in the remaining sections was as follows:

In Section V, Obstetrics, the Causes and the Treatment of Non-puerperal Hemorrhages of the Womb. Reporter, William H. Byford, M. D., Professor of Obstetrics and Diseases of Women in the Chicago Medical College; in Section VI, Ophthalmology, the Comparative Value of Caustics and Astringents in the Treatment of Diseases of the Conjunctiva, and the best mode of applying them. Reporter, Henry W. Williams, M. D., Professor of Ophthalmology in Harvard University; in Section VII, Otology, Importance of Treatment of Aural Diseases in their early Stages, especially when arising from the Exanthemata. Reporter, Albert H. Buck, M. D., of New York; in Section VIII, Sanitary Science, the Present Condition of the Evidence concerning "Disease-germs." Reporter, Thomas E. Satterthwaite, M. D., of New York; in Section IX, Mental Diseases, the Microscopical Study of the Brain. Reporter, Walter H. Kempster, M. D., Physician and Superintendent of Northern Hospital for Insane, Oshkosh, Wisconsin.

In the evening a reception was given to the members of the Congress and their ladies, by the Medical Profession of Philadelphia, in the Judges' Hall, Exhibition grounds. At its conclusion a supper was served in the LaFayette restaurant in the grounds.

Gov. Hartranft and Gen. Hawley, President of the Centennial Commission, were present at the reception.

The Congress met at ten o'clock on Tuesday morning in the Chapel of the University, the President, Prof. Gross, in the chair. After the reading of the minutes, the report from the sections were received, and after some discussion as to the propriety of endorsing their conclusions, they were at last merely referred for publication. Letters of congratulation were read from the St. Petersburg Medical Society and the University of Christiania, Norway. Resolutions were offered by Prof. Flint and adopted by the Congress complimentary to the Surgeon General and staff, and recommending that the U. S. Congress be appealed to for encouragement of the work and the publication of a Catalogue of the National Medical Library. The Committee on Nominations made an additional report, which is comprised in the list of officers in the first day. Dr. Eve, Vice-President, was called to the chair, and Dr. H. I. Bowditch read the address on Hygiene and Preventive Medicine. He divided the past one hundred years into three portions, the era of theory and systems, the age of strict observation and recording of facts, and the period embraced in the last few years, 1869–76, the period in which State Medicine and Hygiene were in the advance.

To determine the present condition of sanitary progress, Dr. Bowditch sent inquiries to one hundred and sixty-seven men of intelligence, living in thirty-eight States and nine Territories, covering twenty-five degrees of latitude and forty-seven degrees of longitude, and occupying every phase of climate. Some of these replies, arranged according to States, may be briefly given:

Does the State duly appreciate and care for the health of the people? 34 nays, 8 yeas.

Is the State willing to spend money to establish a State Board for various beneficent purposes cited? 36 nays, 10 yeas. In some States these boards are established, but no money is appropriated.

Is the State willing to spend money for investigation of preventable causes of disease? 30 nays, 12 yeas. In Massachusetts ten thousand dollars were appropriated last year to check the pollution of streams.

Is the State willing to spend money for the prevention of noxious trades? 26 nays, 14 yeas. Brighton, Mass., once the center of numerous offensive slaughter-houses, is now a beautiful and fashionable spot, since these were got rid of.

Is the State willing to take measures to prevent the adulteration of food? 23 nays, 16 yeas.

Has the State any Board of Health? has it a properly organized set of correspondents? No perfect set exists in any State. There are twelve State Boards of Health altogether.

Has the State a law for the registration of vital statistics, for irrigation, prevention of disease, sanitary survey, etc.? 40 nays, no yeas.

Has it a law for the registration of marriages, deaths, etc.? 16 nays, 20 yeas; eighteen of the latter being qualified, stating that the law is imperfect, a dead letter, carries on in large cities only, etc.

Has it any law in regard to the drainage of lands? 24 nays, 7 yeas; but in the latter the drainage is for agricultural, not sanitary purposes.

Has it a law for introduction of water into cities? 23 nays, 14 yeas; but there is no law to insure the purity of the water.

Has it any law in regard to contagious diseases? 16 nays, 21 yeas. There should be a national law for the enforcement of vaccination, and every foreign nation should adopt it. An international code of health must come sooner or later.

Has it taken any action in regard to the use of well-water, etc? In one hundred and forty-eight towns eighty-two wells were reported, the water in the rest being derived from rivers. Two-thirds of the people in the United States are living in total disregard of all knowledge or interest in the kind of water they are using.

Has it any regulation for the disposal of sewage? Only one-fifth have any definate idea that any action is necessary.

Has the State, county, or city published health reports? Three-fourths of the States ignore the matter.

Is there any known law of development or partial development of disease to give as a legacy to the next century as a preventive? 36 nays and 6 yeas. Special points are mentioned as to the development of zymotic diseases, modes of prevention, etc.

Our present duty is organization. Two hundred thousand persons are annually slaughtered in the States by preventable diseases. The speaker concluded by expressing great hopes of the future, and of the coming century in the increased health, and the lengthening of human life it was sure to bring with it.

At the conclusion of Dr. Bowditch's address, Prof. T. G. Wormley of Ohio, read the Address on Medical Chemistry and Toxicology. Of this we are unable to give an abstract.

In the sections some very interesting papers were read. In the section on Medicine, The unity or duality of croup or diphtheria, the discussion being opened by a paper by Dr. J. Lewis Smith of New York. The conclusions, with which the majority assented, were that they were two distinct maladies. A paper was also read in this section upon Medical Teachings, by Dr. A. P. Reid of Halifax, which was not reported to the Congress.

Dr. Flint, Jr.'s, paper in the section on Biology was upon the Excretory functions of the Liver. We give the following outline furnished to the Committee of Arrangements.

Is the liver, as far as the production of bile is concerned, an organ for secretion, for excretion, or has the bile functions both as a secretion and an excretion? The bile contains one substance, cholesterine, which is evidently separated from the blood by the liver and is not formed in the substance of the liver itself. The blood which goes to the liver contains more cholesterine than the blood which has circulated through this organ. It is evident that cholesterine is produced in certain of the tissues, particularly in the brain and nervous system. The blood gains cholesterine in its passage through the brain. In old cases of hemiplegia, there is no cholesterine in the blood taken from the arm of the paralyzed side, while it exists in the blood from the sound side. In certain cases of structural disease of the liver, cholesterine accumulates in the blood and produces peculiar toxic effects. The same effects follow the injection of cholesterine into the blood of living animals. Cholesterine is an excrementitious substance; it bears the same relation to the liver that urea bears to the kidneys; it is discharged in the bile into the small intestine, is transformed during digestion into another substance (stercorine) and as stercorine exists in the fæces. In addition to the excrementitious function of the bile, this fluid has another function, which latter is connected with digestion and is essential to life.

In the Surgical Section the interest was concentrated in listening to the remarks of Prof. Lister, on Antiseptic Surgery. Owing to the lateness of the hour on Monday, Prof. Lister was unable to enter into a discussion of the paper upon this subject by Prof. Hodgen. The speaker first alluded to the great trouble which was experienced in faithfully carrying out the antiseptic method perfectly, but he did not think there was any medical man who would shrink from any trouble, no matter how great, which was calculated to help his patient, and to hasten recovery. He alluded to several operations whose success would be at least conjectural without the aid of the antiseptic system of operating. He said: "I should be exceedingly sorry to apply any ligature without strict antiseptic treatment." Antiseptic surgery is dealing with surgical cases in such a manner as to prevent putrefaction. During the course of his remarks, Prof. Lister exhibited his common method of dressing. He uses Carbolic Acid, but insists that it shall be perfectly pure. That which makes carbolic acid unpleasant to the smell, he said, was cresyllic acid. In a solution of carbolic acid and water, not perfectly clear, the cloud is caused by insoluble carbolic acid, and it is that which irritates the hands, but a perfectly pure solution will not do this. He is very particular in every detail of the application of his dressing. The finger-nails are carefully cleaned before a finger is introduced into the wound. The instruments are all first dipped in the carbolic solution, and if during the operation one of them is laid upon the table, it is again dipped in the solution before being used. The entire dressing is done under a cloud of spray, and when it is changed it must be under spray. Prof. Lister's remarks were extended to a great length, being about three hours in duration.

In the Section on Dermatology and Syphiology, an interesting paper was read by Dr. L. Duncan Bulkley, of New York, upon the question: Are Psoriasis and Eczema local diseases, or are they manifestations of constitutional disorders? The conclusion reached was in favor of their constitutional origin.

The paper of Dr. Goodell, of Philadelphia, upon the Mechanism of Natural and of Artificial labor in Narrow pelves, and of Dr. Holmes, of Chatham, Ontario, upon the Management of Convulsions in Children, were listened to with interest in the Obstetrical Section, and provoked an animated discussson of the principles they held forth. These papers were followed by a paper upon Enucleation in Ovariatomy, by Prof. J. F. Miner, of Buffalo. Dr. Miner recited the steps by which he had arrived at his present conclusious in regard to Enucleation, explained its principles and adaptation, and urged its trial by members of the section. An interesting discussion followed, in which the merits of the various methods were pretty freely canvassed. The remarks upon the paper were participated in by Drs. Barnes, of London, Simpson, of Edinburgh, Parvin, of Indianapolis, White, of Buffalo, Kimball, of Lowell, Peaslee, of New York, and others.

The remaining sections were not very well attended, the interest of the Congress seeming to centre in those already named, excepting, perhaps, that upon Mental Diseases, in which a paper was read by Dr. Isaac Ray, upon the Responsibility of the Insane for Criminal Acts. This paper resulted in the passage of the following resolution:

*Resolved,* That there is at present manifested a tendency to hold the insane responsible for the commission of acts. That this tendency is unjust, unphilosophical, and contrary to the teaching of pathology, which clearly points out insanity is the expression of disease.

The papers in the other sections were upon the following topics:

In Section VI, Ophthalmology, Tumors of the Optic Nerve. Reporter, Hermann Knapp, M. D., of New York; in Section VII, Otology, What is the Best Mode of Uniform Measurement of Hearing? Reporter, Charles H. Burnett, M. D., Aural Surgeon to Presbyterian Hospital, Philadelphia; in Section VIII, Sanitary Science, Hospital Construction and Ventilation. Reporter, Stephen Smith, M. D., Professor of Orthopædic Surgery in the University of the City of New York.

In the evening two receptions were given by Drs. Thomson and Wilson; they were very elegant affairs and were well attended.

The Congress assembled on Wednesday at ten, A. M. President Gross in the chair. Dr. J. L. Atlee moved that the Secretary of the Publishing Committee be requested to send to the Governor of each State and Territory, and to each Province of Canada, a copy of the address of Dr. Bowditch. Adopted.

A request was received from the National Temperance Association, at New York, asking the Congress to make a declaration in regard to the effect of alcohol as a beverage. The request was laid upon the table. The reports from the sections were received, after which Dr. Seguin, of New York, addressed the Congress upon the subject of Uniformity in Weights and Measures. At the conclusion of his remarks the following resolutions were passed:

*Resolved,* That the International Medical Congress of 1876 recognize the advantages which would accrue from the introduction of a gradual uniformity in the multiple and heterogeneous elements of physic, as posology, nomenclatures, etc., and in the means and records of medical observation.

*Resolved,* That in consequence, this Congress authorizes the President to appoint delegates to the International Congress of 1877, with the special mission of presenting a schedule of the means of uniformity in physic actually applicable in all countries, and another of those which could soon be made acceptable by the profession at large.

*Resolved*, That the said delegates be advised to invite the co-operation of the men who have already worked for the same cause at the International or National Medical or Pharmaceutical Congress of Paris, Vienna, St. Petersburg, and Brussels.

The address on Surgery was then delivered by Prof. Paul F. Eve, of Nashville, Tenn. This address was so much of it historical, that it will be impossible to give anything like a summary of it. The address of Dr. J. M. Toner, of Washington, upon Medical Biography was also of such a character that nothing but the entire paper would be at all satisfactory.

In the section on Surgery, Prof. Lewis A. Sayre, of New York, read a paper on the Treatment of Coxalgia. Dr. Sayre drew the following conclusions: That morbus cexarius is a disease of early childhood; that it is almost always due to traumatic origin and not necessarily connected with a vitiated constitution; that rest of the parts and freedom from pressure, while at the same time the patient is allowed to exercise in the open air, is the best treatment which can be adopted; that if this plan is adopted in the early stages, the majority of cases will recover with nearly, if not quite perfect motion, and without deformity.

In the advanced *second* stage of the disease, when the absorption cannot be produced, it is better to aspirate the joint and remove its contents, than to wait for ulceration and discharge. If proper treatment has been neglected until the bone has become carious, exsection is justifiable and far preferable to the slow process of exfoliation, giving much better results in usefulness of the limb and infinitely better as to deformity of the body and motion of the joint. An animated discussion arose at the conclusion of this paper between Prof. Sayre and Prof. Gross, the latter being inclined to favor the theory that constitutional defects had an active agency in the production of hip joint disease. At the conclusion of Dr. Sayre's paper Prof. Wm. H. Van Buren read a paper upon the Medical and Surgical Treatment of Aneurism. Dr. Van Buren's conclusions were as follows:

1. Tufnell's treatment of aneurism, by rest, position, and restricted diet, offers a valuable resource in thoracic and abdominal aneurisms.

2. It should always be tried in innominate, subclavian, subclavio-axillary, and iliac aneurisms, before resorting to measures attended by risk of life.

3. For aneurisms of the subclavian and iliac arteries, the Hunterian operation, with our present means of preventing secondary hemorrhage, is not justifiable.

4. For reasons formally set forth by Holmes and Henry Lee, the "old operation" cannot properly be substituted for the Hunterian operation in these cases, but should be held in reserve for special cases.

5. It is the most safe and surgical resource in gluteal aneurism, if the circulation can be commanded by the hand *in recto.*

6. The mode of cure by embolism, aimed at in the method of manipulation, is a not unfrequent explanation of what is called spontaneous cure of aneurism.

7. The value of Esmarch's bandage in the treatment of aneurism is probably not fully estimated.

8. In view of the promising features presented by the cases of Levis and Bryant, into which horse-hair was introduced into an aneurismal tumor, the repetition of this operation, or the substitution for horse-hair of Lister's prepared catgut or other animal substances, may be properly tried.

In section one, Medicine, a paper was read by Prof. Roberts Bartholow, of Cincinnati, upon the question, Do the Conditions of Modern Life favor, specially, the Development of Nervous Diseases?

The paper was referred to the Committee on Publication, without any expression of the opinion of the Section upon the questions involved.

Dr. Bartholow's opinion was that the increase in insanity is more apparent than real, its seeming increase being due to the increase of intelligence upon the subject, the spread of news by the press and the increased attention paid to victims of insanity. A paper was also read in this section upon the Etiology of Epilepsy, by Dr. Wm. B. Neftel, of New York, and one by title upon the Treatment of Phthisis Pulmonalis, by Dr. E. G. Eliascopulus of Galaxidi, Greece.

In the section on Biology, Prof. Rudnew, of St. Petersburg, presented four papers, as follows :—On the Structure of the Sweat Glands in Man, the Horse, and Sheep, by Dr. Galani; on the Nerves, and their Termination in the Pleuræ of the Rabbit, the Dog, and the Cat, by Dr. Lebedeff; upon the Miscroscopical

Anatomy of the Nervous Apparatus of the Bronchi and Lungs in the Frog, the Rabit, and the Dog, by Dr. Jantchich ; upon the Endings of the Nerves in Skin of Man, by Dr. Jantchich. Dr. J. G. Richardson read a paper upon the prevention of fungous growths in solutions for Hypodermic injection, and their prevention by Salicylic Acid.

In Section IV, Dermatology and Syphilology, the question assigned for discussion was, The Virus of Venereal Sores ; its Unity or Duality. The reporter was Dr. F. J. Bumstead, of New York. The conclusions which he adopted, and which received the sanction of the Section were as follows :—

1. The virus of venereal sores is dual.
2. Venereal sores may be due to the inoculation of the syphilitic virus, and also to the inoculation of products of simple inflammation.
3. These two poisons may be inoculated simultaneonsly.
4 (additional). The present state of science has demonstrated that suppurating inflammatory lesions resembling chancroids, may be produced on various portions of the body by inoculation with simple pus from varions lesions.

A paper upon the same subject by Dr. C. R. Drysdale, of London, was read by title. Dr. Heitzman, of New York, presented a paper upon Seborrhœa.

In Section V, Obstetrics, Dr. W. L. Atlee introduced the subject of the treatment of Fibroid Tumors of the Uterus.

The subject was treated mainly from the standpoint of personal experience.

Two principal divisions of the subject were made, as :—

I. Tumors usually accompanied with hemorrhage, embracing (*a*), fibroids occupying the vaginal canal ; (*b*), fibroids within the cavity of the uterus ; (*c*), interstitial submucous fibroids ; (*d*), interstial fibroids proper ; (*e*), recurrent fibroids.

II. Tumors usually not accompanied with hemorrhage, including (*a*), interstitial subperitoneal fibroids ; (*b*), sessile peritoneal fibroids; (*c*), pedunculated peritoneal fibroids ; (*d*), interstitial cervical fibroids ; (*e*), myomatous degeneration of the uterus ; (*f*), fibro-cysts of the uterus.

The author dwelt upon the best mode of treatment both surgical and medicinal—the removal of tumors *per vias naturales*—and by abdominal section—the propriety of extirpating a fibroid uterus by either of these methods—a consideration of the several agents which are supposed to control the growth of fibroid tumors.

Following Dr. Atlee's paper were two others, one upon the Three Most Important Obstetrical Instruments by Prof. Lazarewich, of Kharkoff, Russia, and one upon the Cure of Ovarian Cysts by Electrolysis, by Frederick Semeleder, M. D., of Vienna. Dr. Semeleder detailed some very remarkable results and evidently excitedly considerable interest in the subject in the minds of his auditors.

The work in the other Sections was as follows :—

Section VI, Ophthalmology, Orbital Aneurismal Disease and Pulsating Exophthalmia ; their Diagnosis and Treatment. Reporter, E. Williams, M. D., Professor of Ophthalmology in Miami Medical College of Cincinnati ; in Section VII, Otology, in what Percentage of Cases do Artificial Drum-membranes prove of Practical Advantage ? Reporter, H. N. Spencer, M. D., of St. Louis ; an interesting paper by Dr. S. J. Jones, of Chicago, upon Certain Modifications in the Ordinary Methods of Treatment of Chronic Non-Suppurative Inflammation of the Eustachian Tube and Middle Ear was also read in this section. The papers in Section VIII, Sanitary Science, were upon the General Subject of Quarantine with Particular Reference to Cholera and the Yellow Fever. Reporter, J. M. Woodworth, M. D., Supervising Surgeon-General U. S. Marine Hospital Service ; Disinfection in Yellow Fever. By C. B. White, M. D., of New Orleans ; in Section IX, Mental diseases, Simulation of Insanity by the Insane. Reporter, C. H. Hughes, M. D., of St. Louis, Mo. We are able to give the following resumé of Dr. Hughes' paper :—

The feigning of insanity by the sane has long been recognized as a practical fact. The possibility of similar efforts on the part of men really insane has been ignored or forgotten. The fact that the proof of simulation possesses no real practical value, in the case of a person already adjudged to be insane, is, probably, one cause of the rareness of recorded cases.

Advanced general dementia is incompatible with simulation. Acute and general mania is also incapable of coexistence with feigning. In recovery from the latter condition, circumstances might easily give rise to simulation of a state recently passed through. Experience and observation might certainly help to an excellent imitation of a state so lately endured.

Simulation requires and implies some degree of rationality, and usually some motive. This is by no means incompatible with insanity. In the remissions of periodic mania, in certain cases of chronic general mania and certain forms of hysterical mania, and especially in affective or moral insanity without distinct intellectual impairment, simulation is perfectly possible and practicable. The existence of susceptibility to ordinary motives is recognized in the management of every insane asylum.

Striking instances of success in the simulated abandonment of delusions, so common in alienistic literature, suggest an equal facility at invention or pretence.

The criminal classes of our great cities are born and trained to deception. Simulation might very naturally be added to constitutional infirmity. Such cases probably occur oftener than is supposed. Many famous and historic cases might be correctly characterized as con pounds of simulation with actual disease.

Rarely does insanity affect all the faculties alike. Among the rational acts done by the insane man simulation may happen to occur. Especially probable is it that a man recovering from mania might imitate the crazy acts recently prompted by disease if adequate motive existed.

Simulation is peculiarly applicable in those forms of insanity which involve the affective faculties, leaving the intellect comparatively untouched.

The question of responsibility in cases where simulation is mingled with actual disease is a very difficult one. The ancient legal test, "knowledge of right and wrong," is here wholly inadequate.

The motive for simulation in the insane of hysterical tendencies is often the craving for sympathy and attention. Occasionally, however, it seems to be wholly motiveless—a mere freak of disease.

We should beware of inferring because of detected simulation, the non-existence of disease.

At 7. 30 P. M., the Congress was addressed by Dr. J. J. Woodward, Surgeon U. S. Army, upon the Medical Staff of the United States Army, and its Scientific Work. Dr. Woodward's address was delivered in the Hall of the Jefferson Medical College, and was well attended.

He sketched the nature of all the papers and reports connected with the medical service. 200,000 had died of sickness during the war; 100,000 of wounds. 16,000 volumes of manuscript record books, and many tons of papers of all kinds, came back to the Surgeon-General when the hospitals were closed after the war, all of which had to be examined and analyzed. It would have been a great national crime not to have done so, and not to have extracted from them the immense amount of material they contained for scientific and useful purposes. No great national medical library existed at the beginning of the war; nor was there any great pathological museum. Valid reasons for the establishment of each at Washington, under national auspices, were given by the speaker. The whole medical staff of the army had contributed to both, but the heavy labor had been performed by three Assistant Surgeons, Drs. Billings, Otis, and Woodward, without extra compensation, in addition to their other administrative work.

Since 1865, the history of 300,000 cases had been traced through the records of the Surgeon-General's office, but, from want of proper clerical force, 12,000 to 13,000 applications were now lying unanswered, the number of clerks being reduced from 100 to less than half that number. Over 6,000 quarto pages of publications have thus far been issued from this office. The reports on meteorology, formerly published, are now discontinued, the Signal Office having taken charge of this branch of inquiry. Other reports emanating from the Surgeon-General's office were referred to, as that of Dr. Billings on posts and hospitals, the records of physical examination of recruits, the report of Dr. Ely McClellan on Cholera, etc. The importance of the chemical laboratory connected with this office was dwelt upon.

The volumes of the "Medical and Surgical History of the War," were the records of 270,000 cases. Cheney, in his medical statistics of the war of the Crimea, had 40,000 cases to describe, and 17,000 in the Italian war; but they were not subsequently followed up, as they have been with us. The National Medical Library at Washington now contains 40,500 bound volumes and 41,000 pamphlets. 275,000 cards of all the subjects in the books and journals of this library await the hands of the printer. The catalogue *raisonne*, when published, will make five volumes of 1,000 pages each. The Army Medical Museum contains 19,000 specimens, illustrating military surgery and camp diseases during the war. The hope is, to make this also a general pathological museum. It contains 1,500 skeletons and crania; 1,600 crania alone from all countries, with complete measurements by Dr. Otis. This museum, if added to from private sources, would be especially valuable, particularly if they were specimens which have formed the basis of original researches.

Dr. Woodward then gave an exhibition with the magic lantern, of pathological specimens, microphotographic sections, etc. The thanks of the Congress were tendered him for his very interesting address.

The Congress assembled in the Chapel of the University on Thursday, Sept. 7th, at ten A. M. Dr. Gross in the chair. The reports of the Sections were received and accepted.

On motion of Dr. H. I. Bowditch, of Boston, the following resolutions were adopted:

*Whereas*, the work already accomplished by the officers connected with the Bureau of the Surgeon-General of the United States Army, in the establishment of a medical library, and in the preparation of its complete and unique catalogue, in the formation of an anatomical museum, from which important scientific results have already been obtained, and which have been not only a source of honor to these United States, but of value to foreign lands and wherever science is cultivated; and

*Whereas*, This Congress learns with regret that, owing to a lack of sufficient clerical force and of pecuniary means, not only some of the works already in progress has been suspended, but that other work of equal value cannot be undertaken, although ample materials for the same are now lying unused in the Surgeon-General's office; therefore

*Resolved*, That a committee of three be appointed to prepare a memorial to be presented to the Congress of the United States, at the earliest day possible, at its next session, to urge sufficient support to these most important works.

*Resolved*, That it is desirable that said memorial be signed by the President, Vice-Presidents, and Secretary General of this body.

The President appointed Drs. Bowditch, of Boston, Rudnew, of St. Petersburg. and N. S. Davis, of Chicago, as the Committee to prepare this memorial.

Upon motion of Dr. J. P. White, of Buffalo, N. Y., it was ordered that the printed pamphlet containing Dr. Bowditch's paper on Hygiene and Preventive Medicine, be sent to the Presidents of State and Territorial Medical Societies and Sanitary Boards of the United States, and the Societies and Sanitary Boards of the Dominion of Canada.

The address on Obstetrics was then delivered by Dr. Theophilus Parvin, of Indianapolis. Prof. H. Miyake, of Tokio, Japan, in the chair. Dr. Parvin said that American obstetric practice was based upon the English rather than upon the French school. He paid a glowing tribute to the early founders of Obstetric teaching in America, and sketched the advances and discoveries which had been made by Americans. He said that the two greatest glories of America's contributions to gynaecology were the cure of genito-urinary fistula and the removal of the ovarian tumor. Holding them up she could justly say, "these are my offerings to humanity and medicine."

The address on Medical Jurisprudence was delivered by Prof. Stanford E. Chaillé, of New Orleans. He said:

Medical Jurisprudence owes its power to knowledge derived from every branch of medicine, but the law determines how far this power shall be utilized in the administration of justice. Hence the development of medical jurisprudence has varied in different nations with the progress of medical science, and with the extent of its applications to the protection of property, reputation, and life. Efficiency in this legal applicatian varies with the appreciation of medical knowledge by the rulers of a nation, and it results that laws more favorable to the culture of legal medicine are to be found in nations ruled by the educated few than in those governed by the people. The Papal canon laws, originating many medico-legal questions, sowed in 1620 by the hand of Zacchias, a Pope's physician, the first sound seed of medical jurisprudence in the land of Columbus, then the home of science and the arts. The new-born shoot, languishing in Italy, was transplanted to German soil, where it received such culture as nourished its growth, and reproduced seed to generate in other lands.

Since 1790 no nation has surpassed France in the culture of this science. The judges appoint medical experts: these, since 1803, must be graduates in medicine and have passed an examination in legal medicine. How much of medico-legal science has been transported from Germany and France to Great Britian and the United States would, I fear, prove offensive to Gallic and still more to Anglo American vanity. Great Britain transmitted to this nation laws barbarously conspicious for the absence of provisions to apply medical knowledge to the administration of justice, and Anglo-American law continues to be, in large measure, hostile to medical jurisprudence. However, British laws have done something for the science and a little for the art. For Great Britain has fostered medical education; did, in 1803, found a chair of forensic medicine in one university, and has now such chairs in all its medical colleges; has by the registration act and other laws greatly strengthened the medical profession, and has compelled its courts to accept expert evidence only from registered and therefore educated medical men; still "the crowned republic" remains destitute, as does its democratic American offspring, of popular, hence of governmental, appreciation of the legal importance of medical knowledge, as is proved by the same lack of any system to secure the medical evidence of competent experts, as characterized its laws when surgeons were barbers and physicians were astrologers, sorcerers, and interpreters of dreams. What wonder that Germany and France began the study earlier and have prosecuted it more successfully. The States of the Union have for the most part left the culture of medical science to individual enterprise, which supplies solely that which the private citizen demands—practitioners of medicine to heal the sick. The States have as yet made no demand for competent medical experts to aid the administration of justice, and have done nothing designedly for the culture of medical jurisprudence. What growth can this branch of State medicine have so long as a State does not recognize even its existence?

In the United States there are probably forty-five thousand medico-legal autopsies made annually. The services of a skilled expert at these "coroners' inquests" is of inestimable importance. Further, our courts have annually from forty-five hundred to treble this number of criminals necessitating medical testimony. Whatever the number may be, it would indicate inadequately the number of citizens whose welfare is involved in the efficient application of medical knowledge to the administration of justice. What are the methods which the Anglo-American law adopts to secure in practice that "best attainable evidence" which in theory it demands? It entrusts medico-legal autopsies, which require special medical and some legal knowledge, to those having neither the one nor the other, except by accident; for these coroners—whose inexperience our law assumes by constant "rotation of office"—owe their position solely to political popularity, a qualification which a competent expert is most unlikely to possess. Are these unqualified officials supplied with efficient aid? If so, again by accident, since the law leaves it to chance, or to the coroner, or to his still less qualified jury, to provide a medical expert; and, as is usual, accident and ignorance provide inexperience and incompetence. Could ingenuity devise for medico-legal autopsies any methods more inefficient than these, which Anglo-American laws, framed before the birth of medical jurisprudence, have barbarously perpetrated? With the power of medical science thus crippled at the coroners' inquests, then prostituted by the partisan opinions of incompetent experts, then preverted by advocates, and at last, when emasculated of all vigor, submitted for decision to those unable to estimate its weight, what wonder that such gross misapplication of medical knowledge brings upon it that public contempt which justly belongs to methods as monstrous, and to which true medical knowledge is a helpless, pitiable, and disgusted victim. So great has been the diffusion of knowledge, so ardent the love of justice, we have in the main kept pace with, and, in some particulars, outstripped the mother land. Fairly we can name no more, reasonably no more should be expected.

The Sections met at two P. M. In Section I, Medicine, the question for discussion was: "The Influence of High Altitudes on the Progress of Phthisis." Reporter, Charles H. Denison, M. D., of Denver, Celorado.

In Section II, Biology, The Mechanism of Joints. Reporter, Harrison Allen, M. D., Professor of Zoology and Comparative Anatomy in the University of Pennsylvania; in Section III, Surgery, The Causes and Geographical Distribution of Calculous Diseases. Reporter, Claudius H. Mastin, M. D., of Mobile, Alabama; Electrolytic Treatment of Malignant Tumors. By W. B. Neftel, M. D., of New York.

In Section IV, Dermatology and Syphilology, the question was: The Treatment of Syphilis with Special Reference to the Constitutional Remedies appropriate to its various Stages; the Duration of their Use, and the Question of their Continuous or Intermittent Employment. Reporter, E. L. Keyes, M. D.

The following slight modification of the conclusions of the author were adopted by the Section:

Negative conclusions, views for which there would seem to be no foundation in fact.

1. Syphilis commencing mildly needs but little treatment, and does not require mercury.
2. Mercury given internally is necessarily debilitating.
3. Mercury is only useful in secondary syphilis.
4. Iodide of potassium is of considerable value in secondary syphilis.
5. Iodide of potassium is of no value unless preceded by the use of mercury.
6. Iodide of potassium acts by liberating mercury which has been lying latent.

Positive conclusions, which, in the present state of our knowledge, may be affirmed.

1. Mercury is an antidote to the syphilitic poison, and of service in controlling all its symptoms in all, even the latest stages of the diseases; its power over gummata being least, and not to be relied upon.
2. Mercury in minute doses is a tonic.
3. Iodine cures certain symptoms of syphilis, but does not prevent relapses.
4. Mercury, long continued uninterruptedly, so far as practicable in small doses from the time of earliest eruption, constitutes the best treatment of syphilis.

A paper on the same subject by Dr. Charles R. Drysdale, of London, was read by title.

A paper entitled "Verrugas, a Disease peculiar to Peru," by Dr. George A. Ward, of Lima, was also read by title.

The time of the Obstretrical Section was occupied with listening to and discussing the paper of Prof. Wm. T. Lusk, of New York, upon The Nature, Causes and Prevention of Puerperal Fever.

In Section VI, Ophthalmology, the paper discussed was: Are Progressive Myopia and Posterior Staphyloma due to Hereditary Predisposition, or can they be induced by Defects of Refraction, acting through the Influence of the Ciliary Muscle? Reporter, E. G. Loring, M. D., of New York.

The following were the conclusions of the reporter:

From the fact that so large a percentage of children are myopic, whose parents are not near-sighted, while the myopia increases directly with the amount of increased tension of the eyes, and from the fact that an interchange of refraction may occur, whereby an eye which is not congenitally myopic may become so in spite of hereditary tendency against it, it would seem to follow that hereditary predisposition, though undoubtedly a potent cause, is not only not the sole cause, but it is not even the predominating cause.

2. The action of the ciliary muscle, taken by itself, exerts but little influence on the production of myopia, and still less on the formation of the cone.

Dr. Stevens, of Albany, read a paper on the relations between Refractive lesions of the Eye and Corneal Diseases.

The Section on Otology was occupied in the discussion of a paper by Dr. Clarence J. Blake, of Boston, in which the following question was considered; it is one of much importance, and to which, we believe, hitherto, but little attention has been paid: What is the Best Mode of Determining the Hearing of School-Children, and how should partially Deaf Children be Instructed—in mixed classes with those who hear well, or in separate classes where due allowance will be made for their defective hearing?

In Section VIII, Sanitary Science, the Disposal and Utilization of Sewage and Refuse, was discussed. The conclusions reached, were:

1. Every plan for the laying out, or extension, of a city or town, should have as an indispensable part of it, a corresponding and coextensive plan for the continuance or substitution of the natural drainage of the locality, and for the proper construction of a system of sewers.

2. The question in regard to the removal of waste and impurities from towns is not as to the maintenance of sewers, but as to whether they should be depended upon alone, or should be supplemented, more or less largely by other means of conservancy.

3. Every sewer not supplied with a sufficient flow of water to secure the transportation of its contents is a nuisance, intensifying the evils it ought to remove. Ventilation of sewers will mitigate, but not entirely correct such evils.

4. Conditions sufficient for sanitary security are afforded by the discharge of sewage at a considerable distance from a town, into the sea, or into a large and rapid river, whose water, at least for many miles below the exit of the sewers, is not used for drinking.

5. The earth-closet method of removal of excreta is, theoretically and practically, satisfactory in a sanitary aspect; the obstacles to its general adoption belonging only to economy and convenience.

A supplementary proposition, affirming that the sewage-irrigation of arable land, well under-drained, is, where practicable, the most economical method of disposal of sewage, and is free from well-grounded sanitary objections, was not concurred in by the Section, which declined to express an opinion upon that subject, as still open to investigation.

Dr. E. R. Squibb, of Brooklyn, N. Y., read a paper on a "Universal Pharmacopœia."

In Section IX, Mental Diseases, Dr. C. H. Nichols, of Washington, D. C., reported on the best Provision of the Chronic Insane. He said, in substance, that provision for this class of patients should be made by constructing buildings in connection with the several insane asylums. That it is not desirable to construct institutions solely for the care of the chronic insane.

On Friday, Sept. 8th, the Congress again assembled in General Session at ten A. M.

On motion of Prof. Paul F. Eve, of Nashville, the following resolution was adopted:

*Resolved,* That no papers or addresses read before this body, and ordered to be printed, shall be furnished either in abstrrct or otherwise, for publication in any journal prior to publication of the Transactions.

As we have said elsewhere, this motion came too late in the session of the Congress. If the resolution represents the sentiments of those present, they should have assembled with closed doors.

The amount of material on hand being much larger than anticipated, on motion of Dr. Davis, the following preamble and resolutions were adopted:

*Whereas,* This Congress marks an era in the history of medicine in the United States of America, the addresses delivered presenting a summary of the progress in the various departments, which will be of great historical value in all coming time; and

*Whereas,* It is highly probable that these addresses, in connection with the many very valuable papers read and discussed in the Sections, will require for their early publication more money than will be in the hands of the Treasurer for that purpose; therefore,

*Resolved*, That the Committee of Publication be authorized and instructed, as soon as practicable after the final adjournment of the Congress, to ascertain the probable cost of publishing the full transactions in a style appropriate for the work, and if the money on hand is found deficient, they shall address a circular letter to each American member of the Congress, asking for such additional sum, not exceeding ten dollars, for each of such members as will supply the deficiency; and that said Committee be authorized to withhold the volume or volumes, when published, from any member who may neglect or refuse to pay the additional sum required.

*Resolved*, That the Committee on Publication, be authorized and requested to exercise a careful and liberal discretion in preparing and revising the proceedings and reported discussions, in the several Sections, for publication in the transactions of this Congress.

Vice-President McGuire in the chair, Dr. John P. Gray, of Utica, N. Y., delivered the Address on Mental Hygiene. We cannot attempt to give an abstract, as nothing that our brief notes would indicate would do it justice. He referred to the fact that Mental Hygiene had been, until lately, neglected. He considered the topic from three points of view; the individual, the national, and the social. He referred to sociology and political economy as bearing a close relation to the subject. Intellectual activity, he said, is as healthful as physical exercise, but it is to be indulged in under certain restraints and in accordance with certain principles. He referred to the great exhibition, and the influence which the collection of the best exhibits of the arts, manufactures and culture of all nations would have upon mental culture of a people. Education, art, wealth, with all their refining influence, have a marked effect in promoting the mental hygiene of a people. The taste for culture and refinement which they beget, are factors for good which a people or individuals cannot afford to think lightly of. There was a source he said, far above all these, which could not be overlooked. He referred to the dark and stormy times which surrounded this nation at its birth, when its founders were in the habit of looking to God to direct and bless their endeavors. To this and to the God-fearing influence which our ancestors have transmitted to their descendants, is due the dignity and stability of the nation.

He referred to the melancholy example of France, when the peo_ple, in their madness, enthroned a woman as a godess, and worshiped reason as personified in her. He believed that no nation could be substantial which did not have religion as a foundation stone, and what is true of nations is true of individuals. He referred to the motto over the door of the Chapel in which the Congress assembled, " *In Honorem Dei*," and said that that motto was the great secret which men had to learn in Mental Hygiene. Wealth, culture, the highest expressions of æsthetic art, will suffice for the highest development of the national mind, but if underneath all these the people do not recognize the great truths of moral responsibility to the Author and Upholder of all nations, the national as well as the individual life goes out in darkness. " Except the Lord build the house, they labor in vain who build it."

What we have sketched of the drift of Dr. Gray's remarks, will give but a feeble idea of their strength and elegance. In it he took a position which we are glad to see an American bold enough to do in an assemblage of the character of that which he addressed. In our national politics, the truth is becoming painfully manifest that the high moral principles of which Washington's life was an expression, and which actuated and guided his associates, is being lost sight of, and Dr. Gray did well in calling attention to them as one of the principles of Mental Hygiene. Dr. Lunsford P. Yandell followed Dr. Gray in an address upon " Medical Literature." The address was well written and was an interesting historical review of the medical literature of the century. In marked contrast to the address of Dr. Gray, the speaker could not be heard but a few feet from the platform, which was a great fault of some of the other speeches.

In the section on Medicine, Prof. Stillé occupied the chair. A paper on The Treatment of Simple Ulcer of the Stomach, by Dr. H. Lebert, of Breslau, was read by its translator, Dr. Chas. W. Dulles, of Philadelphia. He pursues in his treatment the plan of giving the stomach rest, and employs to that end, nutritious enemata with anodynes. One case under his observation had subsisted for thirty-one days without the introduction of food into the stomach.

Following this paper, Dr. Howard, of Montreal, read a paper on "Progressive Pernicious Anæmia;" a paper was also presented upon "Alcohol in its Therapeutic Relations as a Food and a Medicine." By Ezra M. Hunt, M. D., of Metuchen, New Jersey. The following propositions of Dr. Hunt the Section affirmed and ordered them reported to the Congress in general meeting:

1. Alcohol is not shown to have a definite food value by any of the methods of chemical analysis or physiological investigation.
2. Its use as a medicine is chiefly that of a cardiac stimulant, and often admits of substitution.
3. As a medicine it is not well fitted for self-prescription by the laity, and the medical profession is not accountable for such administration, or for the enormous evils resulting therefrom.
4. The purity of alcoholic liquors is not as well assured as that of articles used for medicine should be. The various mixtures when used as medicine should have definite and known composition, and should not be interchanged promiscuously.

Prof. Rudnew read for Prof. Bérésdow, of St. Petersburg, a paper upon Sclerosis of the Vessels of the Lungs.

In Section III, Surgery, a paper was read by Mr. Wm. Adams, of London, on Subcutaneous Division of the Neck of the Femur, which gave rise to an interesting discussion and the report of other operations of a similar nature.

In the Section on Dermatology and Syphilology, a paper was read by Dr. Engelsted, of Copenhagen, on the Measures to Prevent the Propogation of Venereal Diseases in Denmark. This was followed by a paper by Dr. Drysdale, of London, on The Prevention of Syphilis.

Section V, Obstetrics, Prof. Simpson in the chair. Dr. Fitch, of New York, read a paper on Paracentisis Aspiration and Transfusion. He referred to the methods formerly used in these operations, and in conclusion presented an instrument which he termed the dome-trocar, which he considered admirably adapted to these operations. Dr. Trenholm, of Montreal, then read a paper upon Uterine Hemorrhage, confining his remarks to a form of hemorrhage which he had met with during pregnancy. Following this was a paper by Prof. J. P. White, of Buffalo, upon "Chronic Uterine Inversion." Dr. White explained the claims which he made to priority in this operation over Tyler Smith, and then detailed the reasons for coming to the conclusion which he had reached, that all inverted uteri, of no matter how long duration, were susceptible of restoration, unless bound down by adhesions which it would not be safe to rupture. He related in detail three of his twelve successful cases, and illustrated by diagrams his method of operating. He expressed his belief that there was a stage in the process of uterine involution when it was unsafe to use much if any force, but that when that stage was past the method which he advocated, which was far from forcible, was capable of relieving the deformity. The fundus, except in recent cases, is not "dimpled" in the operation, but that which came down last, i. e. the neck is restored first, then the body, the fundus following. Dr. White's paper elicited an interesting discussion; it was followed by one by Prof. Rochester, of Buffalo, upon Retroversion of the Gravid Uterus, in which he reported a case, and the method he pursued for its relief.

The programme in the other Sections was as follows:

In Section VI, Ophthalmology, Report of One Hundred Cases of Senile Cataract. By Dudley S. Reynolds, M. D., of Louisville, Kentucky; in Section VII, Otology, Aural Vertigo with Variable Hearing. By Charles H. Burnett, M. D., Aural Surgeon to the Presbyterian Hospital, Philadelphia; in Section VIII, Sanitary Science, Metrical System of Weights and Measures. By E. R. Squibb, M. D., Brooklyn, New York; and in Section IX, Mental Diseases, Treatment of Inebriates in Asylums. By George Burr, M. D., of Binghamton, N. Y.

In this Section Dr. Edward Spityka read a paper "On the Methods of Examination which will reveal a Clear and Decisive Connection between Symptoms of Insanity and the Pathological lesions on which they depend."

Friday concluded the work in the Sections. On Friday evening, the Public Dinner of the Congress was given in St. George's Hall. About 170 delegates were present. Prof. Gross presided, supported on his right by Prof. Lister, of the University of Edinburgh, and General Hawley, of the United States Centennial Commission, and on his left by His Excellency the Governor of Pennsylvania, and Mr. Wm. Adams, President of the Medical Society of London.

Replies to toasts were made by Prof. Joseph Lister, Governor Hartranft, Dr. Woodward, U. S. Army, General Hawley, President U. S. Centennial Commission, Prof. Hjort, of Norway, Profs. J. C. Dalton and Stillé, Mr. Wm. Adams, of London, Dr. Hingston, of Montreal, Profs. Davis, of Chicago, and Chaillé, of New Orleans, and Prof. Gross.

The Congress assembled on Saturday, Sept. 9th, at ten o'clock, Prof. Gross in the chair. The Secretary General reported that four hundred and forty-seven delegates had been registered. On motion of Dr. Toner the list was confirmed. Reports from the Sections were received.

A time of mutual congratulation and general good feeling then followed, which was one of the most pleasant features of the Congress.

This was opened by the following resolutions, offered after a speech abounding with complimentary allusion, by Prof. J. P. White, of Buffalo:

*Resolved,* That the officers and trustees of the University of Pennsylvania are hereby tendered our cordial thanks for the very liberal use of their excellent buildings for the meetings of this International Medical Congress.

*Resolved,* That the officers and trustees of the Jefferson Medical College are hereby tendered the cordial thanks of this Congress for the use of their lecture-room for the most interesting lecture of Dr. J. J. Woodward, U. S. A.

*Resolved,* That the Centennial Medical Commission of Philadelphia are hereby tendered the cordial thanks of this Congress for the most excellent manner in which its members have discharged the arduous duties devolved upon them, and by which our pleasuae and profit have been so much enhanced.

*Resolved,* That the president and other officers of the International Medical Congress of 1876 are hereby tendered the cordial thanks of the Congress for the excellent manner in which they have discharged the arduous duties devolved upon them, and by which our pleasure and profit have been so much enhanced.

*Resolved,* That the cordial thanks of the International Medical Congress are especially due to Drs. Thomson, Wilson, and Strawbridge, and to Messrs. H. C. Lea, and J. B. Lippincott, for their generous hospitality.

These were adopted amidst great applause.

Dr. Grant, of Ottawa, eloquently addressed the Congress, and reported the following resolutions, adopted at a meeting of the Canadian delegates to the Congress:

*Resolved,* That the Canadian members of the International Medical Congress desire to express their sense of the great consideration and urbanity with which they have been treated by the officers and members of the Centennial Medical Commission, and beg by this resolution, to tender their warm thanks for the same.

*Resolved,* That the Canadian members of the International Medical Congress most cordially join with the other members of the Congress, in thanking the members and citizens of Philadelphia for the generous hospitality extended to its members throughout the present session.

Prof. Charles J. Hare, of London, then read the following expression of congratulation on the part of the British delegates:

The delegates from Great Britain to the International Medical Congress at Philadelphia, beg to congratulate the President and the several committees on the complete success of the Congress, on the high value of the various addresses presented to it, and on the forward impulse which it has given to the progress of medicine in the widest sense of that word. They desire at the same time to express in the strongest and warmest terms their sense of and their thanks for the unmeasured kindness and courtesy, and the unbounded hospitality with which they have been received on this Centennial occasion, and to add that they will carry back with them a most grateful recollection of that warm right hand of fellowship which has been so warmly extended to them by their brethren of the United States.

The paper was signed on behalf of the delegates by Dr. Hare, late Professor of Clinical Medicine in University College, London; Mr. R. Brudenell Carter, Hunterian Professor of Surgery in the Royal College of Surgeons of England; and Mr. William Adams, President of the Medical Society of London.

On motion of Dr. Bowditch, it was

*Resolved,* That we, as a brotherhood of physicians from the North, South, East, and West of this country, hereby tender to our associates from other lands our most earnest wishes that they may have safe and happy returns to their homes, and we would suggest the hope that they will carry back many pleasant memories of this fraternal meeting now closing, and which has been most appropriately held in this generous and noble city of Philadelphia.

Prof. Sayre, of New York, offered the following resolution, which was seconded in a beautiful little speech by Mr. Carter, of London, and cordially adopted by the Congress:

*Resolved,* That this International Congress request our President, Prof. Gross, to sit for his portrait, and that the Committee of Publication be instructed to have the same engraved and printed in the frontispiece to the volume of our Transactions.

(It is understood that, for sake of correctness, this shall be a photographic likeness, unless otherwise ordered by the Committee.)

The General Secretary presented the preliminary circular of the International Congress of 1877, at Geneva.

The address on "Medical Education," was then delivered by Prof. N. S. Davis, of Chicago, Ill. Dr. Gibbons, of San Francisco, one of the Vice-Presidents in the chair.

At its conclusion, Prof. Gross made some appropriate remarks.

The Congress was then declared adjourned *sine die.*

